# L-arginine abolishes the hypothalamic serotonergic activation induced by central interleukin-1β administration to normal rats

**DOI:** 10.1186/1742-2094-10-147

**Published:** 2013-12-07

**Authors:** Anderson Iuras, Mônica M Telles, Iracema S Andrade, Gianni MS Santos, Lila M Oyama, Cláudia MO Nascimento, Vera LF Silveira, Eliane B Ribeiro

**Affiliations:** 1 Department of Physiology, Universidade Federal de São Paulo, Rua Botucatu, 862 - 2º andar - Vila Clementino, São Paulo, SP 04023-060, Brazil; 2 Division of Applied Statistics, Universidade Federal de São Paulo, Rua Botucatu, 862 - 2º andar - Vila Clementino, São Paulo, SP 04023-060, Brazil

**Keywords:** Brain microdialysis, Food intake, Nitric oxide, Serotonin ventromedial hypothalamus

## Abstract

IL-1β-induced anorexia may depend on interactions of the cytokine with neuropeptides and neurotransmitters of the central nervous system control of energy balance and serotonin is likely to be one catabolic mediator targeted by IL-1β. In the complex interplay involved in feeding modulation, nitric oxide has been ascribed a stimulatory action, which could be of significance in counteracting IL-1β effects.

The present study aims to explore the participation of the nitric oxide and the serotonin systems on the central mechanisms induced by IL-1β and the relevance of their putative interactions to IL-1β hypophagia in normal rats.

Serotonin levels were determined in microdialysates of the ventromedial hypothalamus after a single intracerebroventricular injection of 10 ng of IL-1β , with or without the pre-injection of 20 μg of the nitric oxide precursor L-arginine. IL-1β significantly stimulated hypothalamic serotonin extracellular levels, with a peak variation of 130 ±37% above baseline. IL- 1β also reduced the 4-h and the 24-h food intakes (by 23% and 58%, respectively). The IL-1β-induced serotonergic activation was abolished by the pre-injection of L-arginine while the hypophagic effect was unaffected.

The data showed that one central effect of IL-1β is serotonergic stimulation in the ventromedial hypothalamus, an action inhibited by nitric oxide activity. It is suggested that, although serotonin participates in IL-1β anorexia, other mechanisms recruited by IL-1β in normal rats are able to override the absence of the serotonergic hypophagic influence.

## Introduction

It is well known that chronic inflammatory states are associated with loss of appetite, contributing to muscle and adipose tissue wasting, debilitation of defenses and impaired prognosis [1]. An action of the pro-inflammatory cytokine interleukin-1β (IL-1β) at central nervous system sites has been implicated in the pathogenesis of anorexia [2]. Additionally, there have been reports on a possible participation of brain IL-1β activity in the physiological mechanisms controlling feeding and energy homeostasis [3,4]. These homeostatic mechanisms are complex and include multiple mediators whose interactions are not completely understood [5,6].

There is evidence that the mechanisms of IL-1β anorexia involve interactions of the cytokine with hypothalamic neuropeptides and neurotransmitters of the energy balance control system [7,8]. Among the numerous factors acting at the hypothalamus to influence energy balance, serotonin (5-HT) inhibits food intake and stimulates energy expenditure [5,9]. The demonstrations that 5-HT receptor antagonism or inhibition of 5-HT synthesis attenuated central IL-1β anorexia suggested a serotonergic mediation for this IL-1β effect [10,11].

Central nitric oxide (NO) activity has been implicated in the modulation of food intake, with an orexigenic effect being suggested by the findings that NO-synthase (NOS) inhibition led to hypophagia in mice and rats and that animal models of obesity have a hyperactive brain NO system [12-14]. In a previous study, we used brain microdialysis to address the question of whether the 5-HT system in the ventromedial hypothalamus (VMH) participates in the anorexia induced by IL-1β in obese Zucker rats. We found that 5-HT release was highly stimulated by a single intracerebroventricular (i.c.v) injection of IL-1β, which also significantly inhibited food intake. The importance of the serotonergic activation to IL-1β effect in the obese rats was confirmed by the demonstration that pre-treatment with L-arginine (ARG), the precursor for the synthesis of NO by the NOS, abolished both serotonergic stimulation and the hypophagia induced by IL-1β [15].

The suppression of IL-1β effects by a treatment leading to increased NO production conflicts with the reported higher NOS-2 expression in various brain areas after acute IL-1β in rats [16-18]. On the other hand, other authors have provided evidence for a reciprocal relationship between central NO and 5-HT metabolism [19-21].

The present study aims to investigate the putative functional interactions of the hypothalamic serotonergic system and the NO system and their relevance to the feeding effect of IL-1β in normal rats.

## Materials and methods

### Animals and surgery

The present study was approved by the Committee on Animal Research Ethics of the Federal University of São Paulo. In accordance with the Committee’s guidelines, the number of animals used and their suffering were kept to a minimum.

Since weaning, male lean Zucker rats (Fa/?) were housed five per cage and maintained in controlled conditions of lighting (12:12 h light-dark cycle, lights off at 18:00 h) and temperature (24 ±1°C), with free access to standard balanced chow (4% fat, 22% protein, Nuvital Nutrients, Columbo, PR, Brazil) and water.

At 4 months of age they were anesthetized with ketamine/xylaxine (67/13 mg/kg) and implanted with both a 21-gauge guide cannula aimed at the VMH (A −2.5, L −0.6, and V −6.9 from Bregma) and a 23-gauge cannula aimed at the lateral ventricle (A −0.9, L +1.6, and V −2.5 from Bregma) [22]. The cannulas were secured to the skull with screws and dental cement; the animals were individually caged and maintained with food and water *ad libitum* thereafter. For measurement of the effect of drug treatments on food intake, a separate group of animals received only the i.c.v. cannula. All animals were used after eight days of surgery.

### Measurement of food intake

The animals were fasted for 20 hours and received one of the following i.c.v. treatments (5.0 μL): artificial cerebrospinal fluid (CSF), 20 μg ARG (Sigma), 10 ng IL-1β (IL, Upstate) or 20 μg ARG followed by 10 ng IL-1β (ARG + IL, with a 2-h interval between injections). Pre-weighed food cups were then introduced in the cages and the intake measured after 4 and 24 h. The ARG dose was based on its lack of an effect on 24-h feeding [21] while the IL-1β dose was chosen due to its ability to inhibit feeding [7].

### Microdialysis experiments

A concentric custom-constructed microdialysis probe (2.0 mm of effective membrane length) was inserted through the VMH guide cannula and fixed to it with a small drop of dental cement. Probe construction was detailed elsewhere [23-25]. The animal was connected to a swivel system and the probe perfused overnight, at 1.0 μL/min, with artificial CSF (145 mM NaCl, 2.7 mM KCl, 1.0 mM MgCl_2_, 1.2 mM CaCl_2_, 2.0 mM Na_2_HPO_4_, pH 7.4). Food was removed immediately after probe insertion.

The next morning, the flow rate was adjusted to 2.5 μL/min; 20-min dialysate samples were collected into 10 μL of 0.5 M perchloric acid and immediately injected into a high performance liquid chromatography (HPLC) system. Baseline samples were collected until 5-HT levels were stable, and then i.c.v. injections were started, typically around noon. A mean baseline level (100% value) was calculated by averaging the three basal samples obtained just prior to the i.c.v. injections of 5.0 μL of artificial CSF or 20 μg ARG. After the collection of six 20-min microdialysate samples, all animals received a 10 ng injection of IL-1β. Nine additional 20-min samples were collected.

### HPLC analysis

Dialysate levels of 5-HT and 5-hydroxy-indol-acetic acid (5-HIAA) were measured by HPLC with electrochemical detection. The system (ESA Inc., Chelmsford, MA, USA) consisted of a model 580 pump with two PEEK pulse dampers in series, a 50 μL Rheodyne PEEK sample loop, a 3-μm MD150 C column, a model 5020 guard cell set at 300 mV, a model 5014B analytical cell set at −175 and 150 mV, and a model 5200A detector. The mobile phase consisted of 75 mM sodium phosphate, 1.5 mM octanesulfonic acid, 50 μM EDTA, 100 μL/L triethylamine, and 10% v/v acetonitrile at pH 3.0. The flow rate was 0.6 mL/min. The detection limit for 5-HT was 1.5 pg/50 μL at a signal to noise ratio of 3:1 [25].

### Histological analysis

For verification of probe and i.c.v. cannula positioning, at the termination of the experiments the animal was deeply anesthetized and decapitated. The brain was removed and 50-μm sections were examined under a microscope, following staining with Cresyl Violet. The animals used in the experiments of food intake were injected with 5 μL of Evans Blue through the i.c.v. cannula and dye distribution was examined. Only the animals in which the correct placements of dialysis membrane and i.c.v. cannula were confirmed were included in the data analysis.

### Data analysis

The results are shown as means ± S.E.M. The 5-HT and 5-HIAA data were submitted to two-way repeated measures ANOVA followed by the Tukey *post hoc* test. Food intake results were compared by ANOVA for independent measures and Tukey test. Significance was set at *P* <0.05.

## Results

### Effect of the i.c.v. treatments on 4-h and 24-h food intake

In comparison to CSF treatment, the injection of IL-1β significantly reduced both the 4-h and the 24-h intake. Pre-treatment with ARG did not modify IL-1β hypophagia. The injection of ARG alone increased the 4-h intake but not the 24-h intake (Table 1).

**Table 1:** Food intake during the 4 hours and the 24 hours after an i.c.v. injection of artificial cerebrospinal fluid (CSF, n = 10), 20 μg of L-arginine (ARG, n = 8), 10 ng of interleukin-1β (IL-1β, n = 10), or 20 μg L-arginine followed 2 h later by 10 ng of interleukin-1β (ARG + IL-1β, n = 8)

	**Food intake (g/100 g b.w.)**		
**Treatment**	**4 hours**	**24 hours**	**Body weight**
**CSF**	1.87 ±0.09	7.88 ±0.45	329 ±8
**ARG**	2.56 ±0.17*	8.50 ±0.51	348 ±9
**IL-1β**	1.44 ±0.17*	4.98 ±0.96*	317 ±7
**ARG + IL-1β**	1.51 ±0.11*	5.13 ±0.22*	326 ±7

*P <0.05 vs. CSF treatment.

### 5-HT and 5-HIAA levels in VMH microdialysates

**Figure 1:**
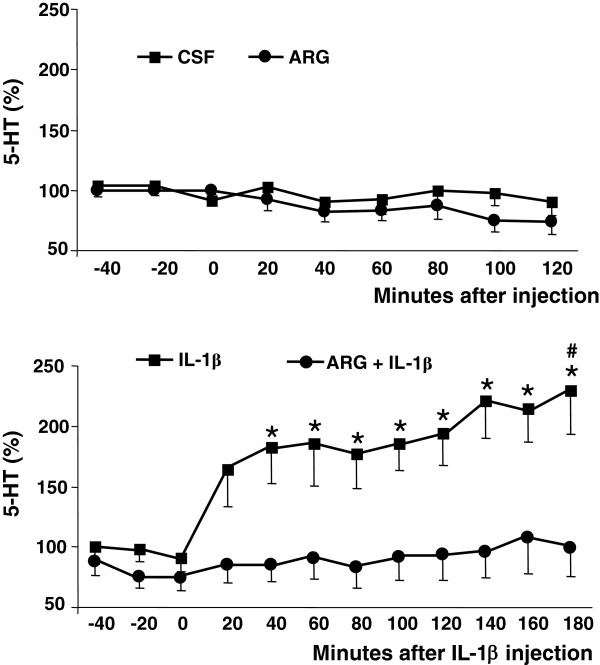
**Effect of ARG and IL-1β on hypothalamic 5-HT levels.** Serotonin levels, expressed as % of the mean basal level, in 20-min VMH microdialysate samples. Upper Panel: Samples collected before or up to 120 min after the i.c.v. injection of 20 μg of L-arginine (ARG, n = 8) or vehicle (CSF, n = 9). Values are means ±S.E.M. Lower Panel: Samples collected before or up to 180 min after the i.c.v. injection of 10 ng of interleukin-1β, in animals pretreated with 20 μg L-arginine (ARG + IL-1β, n = 8) or not (IL-1β, n = 9). Values are means ±S.E.M. *P <0.05 compared with the respective pre-injection levels. *# P* <0.05 compared with ARG pre-treatment.

Mean basal levels of 5-HT and 5-HIAA in the VMH microdialysates were 2.36 ±0.40 pg/50 μL and 230 ±0.06 pg/50 μL, respectively. The treatments with either vehicle or ARG failed to cause significant variations in serotonin levels (F_(8, 120)_ = 1.324, *P* = 0.238) and there was no significant time-treatment interaction (F_(8, 120)_ = 0.748, *P* = 0.649) (Figure 1, upper panel).

Treatment with IL-1β had a significant interaction with sampling time (F_(11, 165)_ = 5.885, *P* <0.001). The injection of IL-1β in the animals not pre-treated with ARG evoked significant increments of 5-HT microdialysate levels. The values were significantly increased from baseline from the second to the ninth sample collected after IL-1β injection. On the other hand, the injection of IL-1β after the ARG pre-injection failed to significantly affect 5-HT levels (Figure 1, lower panel).

**Figure 2:**
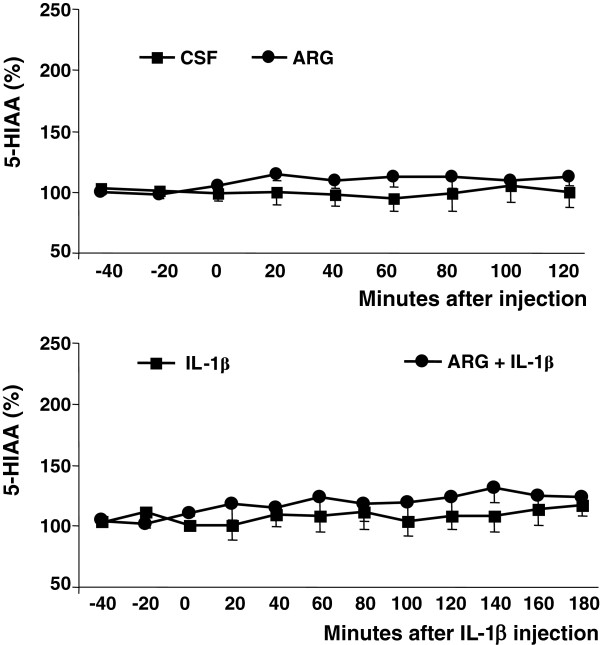
**Effect of ARG and IL-1β on hypothalamic 5-HIAA levels.** Levels of 5-hydroxy-indol-acetic acid, expressed as % of the mean basal level, in 20-min VMH microdialysate samples. Upper Panel: Samples collected before or up to 120 min after the i.c.v. injection of 20 μg of L-arginine (ARG, n = 8) or vehicle (CSF, n = 9). Values are means ± S.E.M. Lower Panel: Samples collected before or up to 180 min after the i.c.v. injection of 10 ng of interleukin-1β, in animals pretreated with 20 μg L-arginine (ARG + IL-1β, n = 8) or not (IL-1β, n = 9). Values are means ±S.E.M.

5-HIAA levels did not show any significant variations during the 2 h following the injection of CSF or ARG (F_(8, 120)_ = 0.719, *P* = 0.67) and no significant time-treatment interaction was detected (F_(8, 120)_ = 1.835, *P* = 0.077). The injection of IL-1β failed to modify 5-HIAA levels (F_(11,165)_ = 1.275, *P* = 0.242) with no time-treatment interaction F_(11,165)_ = 0.764, *P* = 0.675) (Figure 2).

## Discussion

The present data confirmed the ability of IL-1β to inhibit food intake when injected into the central nervous system [7,15,26,27]. This finding reinforces the idea that IL-1β anorexia relies on central actions of the cytokine. We performed a series of experiments aimed at contributing to the understanding of the mechanisms used by IL-1β.

Peripherally-administered IL-β has been shown to induce neuronal activation at hypothalamic sites important to the central control of energy homeostasis, such as the arcuate and the paraventricular nuclei of the hypothalamus [28,29]. Hypothalamic neurons are densely innervated by serotonergic terminals from dorsal raphe nucleus (DRN) and 5-HT released at hypothalamic sites is recognized as an important feeding inhibitory influence [5,9]. We presently show that an i.c.v. injection of IL-1β in normal rats had a potent stimulatory effect on 5-HT extracellular levels in the ventromedial hypothalamus, as evaluated in microdialysis samples. The absence of parallel increments in the dialysate levels of 5-HIAA indicates that stimulation of 5-HT synthesis probably did not contribute to the stimulation of dialysate levels.

Available evidence on the effect of IL-1β upon central serotonergic activity is controversial. Inhibitory effects have been reported, as the cytokine has been found to decrease the firing rate of DRN neurons *in vitro*[30] and to activate the 5-HT transporter in both DRN neurons and striatum terminals [31]. However, stimulatory actions have also been described, such as increased serotonergic transmission in the DRN [32] as well as stimulation of hypothalamic 5-HT turnover and release by IL-1β [33-35]. The present finding is in line with this latter evidence and agrees with our previous observations in obese rats [15].

An important aspect addressed in the present work was the influence of the acute treatment with the NOS substrate ARG on hypothalamic 5-HT. Although a single i.c.v. injection of ARG alone failed to modify the basal levels of 5-HT and 5-HIAA in the microdialysates of the ventromedial hypothalamus, the IL-1β-evoked stimulation of hypothalamic 5-HT levels was prevented by the pre-treatment with ARG. This agrees with other demonstrations of serotonergic suppression after activation of brain NO and of serotonergic stimulation by NOS inhibition in various brain areas [36,37]. Likewise, a single intraperitoneal injection of the inhibitor of NOS, NG-nitro-L-arginine, increased 5-HT metabolism in the diencephalon [19,20].

However, there are also reports showing divergent consequences of acute manipulations of brain NO activity upon the serotonergic system. For example, the administration of ARG into the medial pre-optic area increased local levels of 5-HT [38]. The NOS inhibitor N-nitro-L-arginine methylester failed to affect the increased hypothalamic 5-HT metabolism induced by IL-6 in mice [39] and inhibition of NOS has been found to increase brain serotonergic activity and metabolism while enhanced NO activity has been accompanied by decreased 5-HT levels [40,41]. A possible explanation for these discrepancies could arise from the observation that 5-HT showed a biphasic response to NO donors, with dosages in the low range increasing 5-HT activity while high doses diminishing it [42]. However, an opposite dual relation has also been found, with low doses of NO donors decreasing and high doses increasing 5-HT outflow [43]. Since the ARG dosage used in the present study failed to modify 5-HT levels *per se*, it is indicated that the participation of the NO system on the mechanisms of the serotonergic stimulation induced by IL-1β probably relies on complex interactions with other mediators.

Different lines of evidence suggested the participation of hypothalamic 5-HT on the hypophagia evoked by IL-1β. VMH serotonergic activity was increased in tumor bearing or IL-1β peripherally-treated rats and the local administration of a 5-HT antagonist attenuated hypophagia in these animals [9,35]. Central IL-1β anorexia has also been attenuated by the peripheral treatment with 5-HT2C receptor antagonists, 5-HT1A auto receptor agonists or an inhibitor of 5-HT synthesis [10,11]. IL-1β hypophagia has been associated with activation of melanocortinergic neurons in the paraventricular nuclei [29]. This is in agreement with the previous findings concerning the importance of 5-HT to IL-1β anorexia, since the hypothalamic melanocortinergic system has been shown to mediate serotonergic drug-induced hypophagia [44].

It is thus conceivable that, in the present experimental settings, the serotonergic activation evidenced in the VMH by the microdialysis technique, represented a mechanism favoring IL-1β-evoked feeding-inhibition. However, the results also indicate that additional mechanisms were involved, since hypophagia was still present when the 5-HT increase was prevented. Other mediators of the hypothalamic circuitry regulating energy homeostasis probably contributed to the maintenance of IL-1β hypophagia during serotonergic inhibition, as the cytokine reportedly inhibited orexigenic neuropeptide Y neurons while it stimulated anorexigenic melanocortinergic neurons [35]. This suggestion is consistent with our earlier observation that, in obese Zucker rats, the hypophagia induced by IL-1β was totally dependent on the VMH serotonergic stimulation [15]. We suggested that the absence of 5-HT could not be overcome by the recruitment of other mechanisms during IL-1β hypophagia, unlike the normal rats of the present experiments.

Besides 5-HT, multiple central mechanisms have been implicated in hypophagia induced by IL-1β, including inhibition of glucose-sensitive neurons in the lateral hypothalamic area and stimulation of glucose-responsive neurons in the VMH [7,8]. Hypothalamic neuropeptide systems have also been shown to be targeted by IL-1β. Stimulation of anorexigenic peptides is one important action of the cytokine leading to feeding inhibition. There are demonstrations that corticotropin-releasing hormone, arginine vasopressin, oxytocin, encephalin, cholecystokinin, and proopiomelanocortin, the precursor of alpha melanocyte-stimulating hormone, all play a part as IL-1β downstream effectors. Additionally, IL-1β reportedly inhibited the orexigenic mediators neuropeptide Y and *agouti*-related protein [7,8,18,45,46]. These data indicate that a complex interplay of mediators underlies IL-1β hypophagia and support the present suggestion that one or more of these actions accounted for the maintenance of the hypophagic effect of IL-1β, after the serotonergic inhibition induced by ARG. Which ones were more effective cannot be ascertained at present.

**Figure 3:**
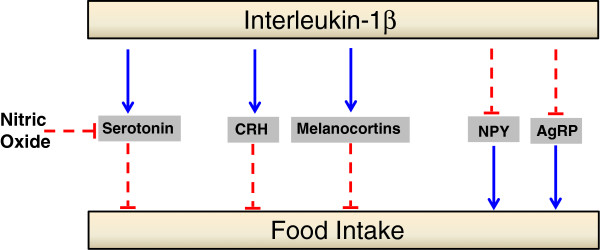
**Schematic of potential central interactions involved in IL-1β hypophagia, showing stimulation (solid arrows) of anorexigenic mediators and inhibition (dashed arrows) of orexigenic mediators.** In normal rats, loss of the serotonergic influence, as induced by nitric oxide stimulation (present results), does not affect IL-1β hypophagia, while the loss of serotonin is not compensated in obese rats, leading to abolition of IL-1β hypophagia [15].

There are reports of impairment of multiple feeding-control systems in obesity, not only in the Zucker strain, but also in other rodent models and in humans [5,23,47-51]. The Zucker mutation leads to severe leptin resistance due the production of an inactive hypothalamic leptin receptor. The blunted leptin action induces upregulation of neuropeptide Y and *agouti*-related protein and downregulation of proopiomelanocortin, cocaine-amphetamine-related peptide, and corticotropin-releasing hormone, among other defects [49-51]. In this setting of multiple disruptions of feeding-regulatory systems, it is not surprising that the serotonergic inhibition could not be compensated in obese Zucker rats, thus abolishing IL-1β hypophagia [15]. Figure 3 shows an schematic of potential central interactions involved in IL-1β hypophagia.

In summary, the present data showed that one central effect of IL-1β is serotonergic stimulation in the VMH, an action inhibited by NO activity. It is suggested that, although 5-HT participates in IL-1β anorexia, other mechanisms recruited by IL-1β in normal rats are able to override the absence of the serotonergic hypophagic influence.

## Abbreviations

ARG: L-Arginine; CSF: Cerebrospinal fluid; DRN: Dorsal raphe nucleus; 5-HIAA: 5-hydroxy-indol-acetic acid; 5-HT: Serotonin; i.c.v.: Intracerebroventricular; IL-1β: Interleukin-1β; NO: Nitric oxide; NOS: NO-synthase; VMH: Ventromedial hypothalamus.

## Competing interests

The authors declare that they have no competing interests.

## Authors’ contributions

AI, MMT and IS performed the experiments and participated in data analysis and manuscript writing. GMSS gave statistical advice. LMO, CMON and VLFS reviewed and edited the manuscript. EBR supervised the experiments and data analysis and wrote the manuscript. All authors read and approved the final manuscript.
